# Intramuscular vitamin A injection in newborn lambs enhances antioxidant capacity and improves meat quality

**DOI:** 10.3389/fvets.2023.1272874

**Published:** 2023-12-04

**Authors:** Pengkang Song, Guoqiang Huo, Jinxin Feng, Weipeng Zhang, Xuying Li, Junxing Zhao

**Affiliations:** College of Animal Science, Shanxi Agricultural University, Taigu, Shanxi, China

**Keywords:** vitamin A, retinoic acid, antioxidant activity, meat quality, longissimus dorsi, lamb

## Abstract

**Introduction:**

Vitamin A (VA) and its metabolite, retinoic acid (RA) possess several biological functions. This report investigated whether neonatal intramuscular VA injection affected antioxidative activity and meat quality in longissimus dorsi (LD) muscle of lambs.

**Methods:**

Lambs were injected with 0 (control) or 7,500 IU VA palmitate into the biceps femoris muscle on day 2 after birth. At 3, 12, and 32 weeks of age, blood samples were collected in the jugular vein for serum levels of RA and muscle samples were collected in the biceps femoris for analysis of relative mRNA expression of enzyme contributors to retinoid metabolism. All animals were harvested at 32 weeks of age and muscle samples were collected to explore the role of VA on the meat quality and antioxidant capacity of lambs.

**Results and discussion:**

Our results indicated that VA increased the redness, crude protein, and crude fat (*p* < 0.05), without affecting moisture, ash, and amino acid composition in LD muscle (*p* > 0.05). In addition, VA increased catalase (CAT) activity and decreased malondialdehyde (MDA) levels in LD muscle (*p* < 0.05). Meanwhile, greater levels of CAT and NRF2 mRNA and protein contents with VA treatment were observed in LD muscle (*p* < 0.05), partly explained by the increased level of RA (*p* < 0.05). Collectively, our findings indicated that VA injection at birth could improve lamb meat quality by elevating the redness, crude protein, crude fat, and antioxidative capacity in LD muscle of lambs.

## Introduction

Meat and its related products are important sources of nutrition for human beings. Meat quality is important to ensure consumer satisfaction, which is affected by genetic factors, feeding methods, and the nutritional status of the animal (1). Traditionally, human sensory perception is used as a factor in evaluating meat quality, including appearance, color, flavor, texture (especially tenderness), juiciness/holding power, and odor. Moreover, freshness or health is also an important quality indicator that is related to the safety of meat for consumption (2). In recent years, demand for lamb meat has consistently increased in several regions of the world (3). Given the higher contents of in iron and zinc (3, 4), spoilage and color change are more easily to be happened in lamb meat (5). The oxidation of lipids and proteins is the main reason for the decrease in nutritional value and the deterioration of sensory and physicochemical properties (such as color, flavor, or tenderness) of meat (6, 7). Particularly, lambs kept in captivity for long periods of time and fed a single variety of feed further exacerbates oxidative stress in the animals and ultimately leads to poor lamb meat quality (8). Therefore, improving the antioxidant capacity of lambs and meat quality has become a priority in meat sheep farming.

Numerous studies have investigated the biological functions of VA as a natural nutritional supplement, where antioxidant capacity (9), adipogenesis (10), myogenesis (11), mitochondrial function (12), and the regulation of muscle fiber type conversion (13) may be associated with improved meat quality. When VA enters the cell, it produces RA under the action of dehydrogenase. In the nucleus, RA binds to the heterodimer of retinoic acid receptor (RAR) and retinoid X receptor (RXR) to perform its biological function (14).

Indeed, VA as a natural antioxidant was reported as early as 1932, and their report indicated the antioxidant potential of VA and carotenoids, both of which protect lipids from rancidity (9). Retinol is a potent peroxy radical scavenger by inhibiting peroxidation in a homogeneous solution of methyl linoleate and in model phosphatidylcholine liposomes (15). Moreover, in previous study, we have shown that neonatal intramuscular VA injection effectively promoted the muscle growth of lambs (16). However, there are no relevant studies to clarify whether there is a potential relationship between VA and lamb meat quality. Traditionally, VA has been added to animal diets as a nutritional supplement (17). However, VA is not completely absorbed by livestock and does not perform its biological functions with this approach. In this experiment, we injected VA into the muscle to ensure that the muscle receives sufficient VA, and the effect is more direct. Thus, the objective of this research was to explore the effects of neonatal VA injection on lamb meat quality and antioxidant activity.

## Materials and methods

### Animal management

All experimental procedures were approved by the Institutional Animal Care and Use Committee of Shanxi Agricultural University (sxnd202028). Randomly selected 80 purebred Hu sheep with similar body states, all of them were on their third pregnancy. In order to avoid the influence of the sire, all ewes were artificially inseminated with semen from one Dorper ram after simultaneous estrus. Attention should be paid to operation and hygiene during insemination. After ewe pregnancy, every 3 pregnant ewes were put into one eweshed. Ewes were fed in the stalls where they were given clean water to drink, and were free to move around after feeding. The diet was formulated to consistent with National Research Council (18) requirements for the nutrition of ewes. During the experimental period, the feeding management and environment of ewes in each group were maintained at the same level and sterilized regularly to keep the eweshed dry and hygienic. At 35 d of gestation, fetal number was determined using an ultrasound monitor. For the follow-up study, only ewes carrying 2 fetuses were used. After birth, we selected twin lambs, both male, weighing 3.5 ± 0.5 kg, and distributed them to control and VA groups to ensure the same body condition of lambs (*n* = 8 in each group).

Based on previous studies (13, 19), we determined to inject 7,500 IU VA palmitate (product no. PHR1235, Sigma, Milwaukee, US) or an equivalent volume of corn oil (product no. c8267, Sigma, Milwaukee, US) into the biceps femoris muscle on day 2 after birth. The lambs were given weekly injections at a fixed point in time for 3 weeks, and paired with ewes for management. Lambs weaned at 12 weeks of age and fed a backgrounding diet for 55 days, followed by a finishing diet with free access to clean water and salt blocks. Moreover, grass hay (peanut seedlings) was added to promote lamb growth during the finishing period. The nutrient composition of concentrate feed and grass hay has been reported in another manuscript (16).

For peanut seedlings, the AOAC method (20) was used to determine DM content, and the Van Soest method was used to determine NDF and ADF contents (21). The Kjeldahl and Soxhlet extraction methods were used to measure the content of crude protein and crude fat, respectively (22). The content of total ash in samples was measured after 40 min of carbonization in a constant temperature crucible at 600°C. All animals were harvested at 32 weeks of age.

### Serum and muscle biopsy collection and treatment

Blood samples of lambs were collected from the jugular vein in the 3rd, 12th, and 32nd week. The serum was separated and stored at −80°C to prevent the degradation of the components in the serum. After determining the detection method, RA in serum was detected by high performance liquid chromatography (HPLC) with a reversed-phase column. (Atlantis^®^ dC18, 5 μm, 100 A, United States), and its content was determined from the standard curve of the standard sample (#B21287, Shanghai, China). The method was as follows: methanol was used as mobile phase with a flow rate of 1.0 mL/min, detection wavelength 325 nm, column temperature 35°C, injection 20 μL (23). Furthermore, the content of Hexokinase (HK) and Lactate dehydrogenase (LDH) in serum and the activities of total antioxidant capacity (T-AOC), total superoxide dismutase (T-SOD), catalase (CAT), glutathione peroxidase 4 (GPx4) and the content of malondialdehyde (MDA) in LD muscle were detected by kits. Information on the kits was given below: HK (#A077-3-1), LDH (#A020-2-2), T-AOC (#A015-2-1), catalase (#A007-1-1), Gpx4 (#H545-1-1), SOD (#A001-3-2), and MDA (#A003-1-2) were acquired from Nanjing Jiancheng Bioengineering Institute (Nanjing, Jiangsu, China).

Skeletal muscle samples were taken from lambs at 3 and 12 weeks of age using a biopsy needle. A small area of wool was removed from the biceps femoris muscle of the lamb’s right hind leg with a shaver and wiped with iodophor. The lambs were fixed and the muscle was removed by inserting a biopsy needle into the biceps femoris and placed in liquid nitrogen for RNA extraction (q-RT-PCR).

### Meat quality analysis

After harvest, the LD muscle of lambs was removed and analyzed for pH, meat color, conductivity, shear force, cooking loss, and drip loss. The pH was measured 45 min after the lamb stopped breathing (pH 45 min) and again 24 h after stopping breathing (pH 24 h) using a pH-STAT meter (SFK-Technology, Denmark). Flesh color (L*, a*, and b*) and conductivity were measured simultaneously with pH, using a carcass conductivity meter and a LAB flesh color meter (Matthäus, Klausa, Germany). For drip loss analysis, the muscle with the fascia removed was trimmed into strips, weighed, and hung on hooks. The strips were placed in a 50 mL centrifuge tube, avoiding contact between the samples and the wall of the tube, and stored at 4°C for 48 h; the strips were removed, and the water on the surface of the strip was absorbed with filter paper and each strip was weighed. For the determination of shear force, the muscle sample was aged at 4°C for 72 h, then exposed to a thermostatic water bath at 80°C and heated until the central temperature of the muscle reaches 70°C. Muscle samples of 3 cm in length and 1 cm^2^ in cross-section were cut from samples cooled to room temperature in the direction of the muscle fibers and shear force, expressed in “N,” was measured using a shear gauge (Mecmesin, West Sussex, United Kingdom). Approximately 100 g of muscle was weighed for measurement of cook loss. The method was as follows: the sample was stripped of its outer membrane and attached fat, weighed and steamed in boiling water for 30 min. The cooked muscle samples were hung in a cool place for 30 min and weighed.

### Meat nutrient analysis

Recognized AOAC methods (24) were used to analyze the moisture, crude protein, ether extract and total ash contents of LD muscle. Specifically, moisture content was calculated by baking constant weight samples at 105°C. The Kjeldahl and Soxhlet extraction methods were used to measure the contents of crude protein and ether extract, respectively, (22). The content of total ash in samples was measured after 40 min of carbonization in a constant temperature crucible at 600°C.

### Muscle periodic acid–Schiff staining and glycogen analysis

The muscle tissue samples were fixed in 4% PFA for 2 days, dehydrated serially by gradient ethanol and xylene, then immersed in paraffin for 9 h, and embedded in paraffin. Then, a microtome (Leica, Germany) was used to slice the samples to a thickness of 5 μm and sections were sequentially dewaxed, rehydrated and stained in periodic acid–Schiff (PAS) staining solution (cat no. G1008, Servicebio, Wuhan, China), then rinsed in running water, dehydrated, transparent, sealed, and finally observed with a microscope (DMi8 microscope, Leica, Germany) for imaging acquisition and analysis (All muscle tissue samples were cut at intervals of 50 μm, with at least 3 replicates in each sample). To measure glycogen content, LD muscle samples were grinded and homogenized in 0.9% saline by a homogenizer, centrifuged (2,500 × g, 10 min, 4°C) and the supernatant was extracted to measure the glycogen concentration with a kit (#A043-1-1, Nanjing Jiancheng Bioengineering Institute, Jiangsu, China).

### Muscle Masson staining and collagen analysis

Muscle samples were embedded in optimal cutting temperature (OCT) compound and sliced into 10 μm thickness sections with cryostat microtome (Leica, Germany). Frozen sections were sequentially dewaxed, rehydrated and stained in Masson staining solution (cat no. G1006, Servicebio, Wuhan, China), then rinsed, dehydrated, sealed, finally observed with a microscope (DMi8 microscope, Leica, Germany) for imaging acquisition and analysis (All muscle tissue samples were cut at intervals of 50 μm, with at least 3 replicates in each sample). Moreover, we analyzed the relative expression of type I, type III collagen fibrils and fibronectin.

### Amino acid composition

Determination of amino acids contents in LD muscle of lambs using high performance liquid chromatography (HPLC). The method was as follows: mobile phase (A: CH₃COONa; B: CH_3_OH, H_2_O), flow rate 1.0 mL/min, detection wavelength 360 nm, column temperature 40°C, injection 20 μL. The amino acid content in the LD was determined from the standard curve based on the standard sample (AAS18-10ML, Sigma, Milwaukee, United States).

### Quantitative real-time PCR

Total RNA was extracted from muscle tissue using Trizol reagent (Sigma, Saint Louis, MO) and synthesized into cDNA using a reverse transcription kit (TAKARA Co, Ltd., Dalian, China). The CFX RT-PCR detection system (Bio-Rad, Hercules, CA) and SYBR Green RT-PCR kit (TAKARA Co, Ltd.) were used for q-RT-PCR. The procedure was as follows: 95°C, 10 min; 45 2-step cycles of 95°C, 15 s, 60°C, 30 s, with at least 3 replicates per set. Primer sequences are shown in Table 1. The relative changes of gene expression were calculated by 2^−ΔΔCt^ method (8), and β-actin was used as house-keeping gene. Gene expression in the VA group showed a fold change compared to the control group.

**Table 1:** Primer sequences for real-time PCR.

Gene name	Sequence (5′ → 3′)	Product size, bp
*ADH4*	AGAAAATGGGCACCAAGGGA	80
	ATTCATTGCTGAGGGGCTTGT	
*ALDH1A1*	CGCAACCGAGGAGAAACTCT	200
	TCATAGCCTCCATTGTCGCC	
*ALDH1A2*	AGCTCTGTGCTGTGGCAATA	101
	GTGGAAAGCCAGCCTCCTTG	
*ADH1C*	TCGCTCTGGAAAGAGTGTCC	167
	TGGAAAGCTCCCATGTGCAA	
*SOD*	GGAGACCTGGGCAATGTGAA	182
	CCTCCAGCGTTTCCAGTCTT	
*CAT*	GAGCCCACCTGCAAAGTTCT	148
	CTCCTACTGGATTACCGGCG	
*GPx4*	TCGCTGCTGGCTATAACGTC	189
	GACCATACCGCTTCACCACA	
*NRF2*	TGTGGAGGAGTTCAACGAGC CGCCGCCATCTTGTTCTTG	88
*Collagen I*	GACATCCCACCAGTCACCTG	161
	GGGACTTTGGCGTTAGGACA	
*Collagen III*	AGGGCAGGGAACAACTGATG	145
	ACAGTGGGATGAAGCAGAGC	
*Fibronectin*	ACCCTGGGTATGACACTGGA	165
	CATTCGGCGGATACGGTCTT	
*β-actin*	CGGCTTTCGGTTGAGCTGAC	159
	GCCGTACCCACCAGAGTGAA	

### Western blotting

The muscle samples were ground in liquid nitrogen and lysed with RIPA lysate (1% NaF, 1% Na_3_VO_4_, 1% PMSF, 2% β-mercaptoethanol, 0.1% protease inhibitor, 1 × loading buffer constant volume to 10 mL) for 30 min, then boiled at 100°C for 10 min, centrifuged at 12,000 × g for 8 min at 4°C and the supernatant removed as the isolated protein. Extracted proteins were separated by SDS-PAGE (room temperature, 80 V for 0.5 h, 120 V for 1.5 h) and then transferred to nitrocellulose membranes (4°C, 100 V for 2 h), blocked with 5% skimmed milk powder (Shanghai Sanger Biotechnology Co., Ltd., Shanghai, China) for 1 h. Finally, nitrocellulose membranes were incubated with the primary antibody (4°C, overnight) and the corresponding secondary antibody (room temperature, 1 h). The Odyssey infrared imaging system was used to visualize protein bands, and the band density was standardized to β-tubulin content.

Antibodies against SOD (no. sc-8637), GPx4 (no. sc-50497), and catalase (no. sc-34281) were purchased from Santa Cruz Biotechnology, Inc. (Santa Cruz, CA, United States). NRF2 (no. bs-1074R) and β-tubulin (bsm-33034 M) were from Biosynthesis Biotechnology Co., Ltd. (Beijing, China). A goat anti-rabbit secondary antibody (926–32,211) was from LI-COR Biosciences (Lincoln, NE) and a donkey anti-goat secondary antibody (no. D110120) was purchased from Sangon Biotech Co., Ltd. (Shanghai, China).

### Statistical analysis

Graphpad Prism 9 software (Monrovia, CA, United States) was used for statistical analysis. The lambs used in the experiment were not completely randomly assigned, so we used paired *t*-test to analyze the data. Results were shown as the Mean ± SEM. *p* < 0.05 was considered significant between control and VA groups.

## Results

### Retinoic acid content in serum and retinol metabolizing enzymes mRNA expression in longissimus dorsi muscle

**Figure 1:**
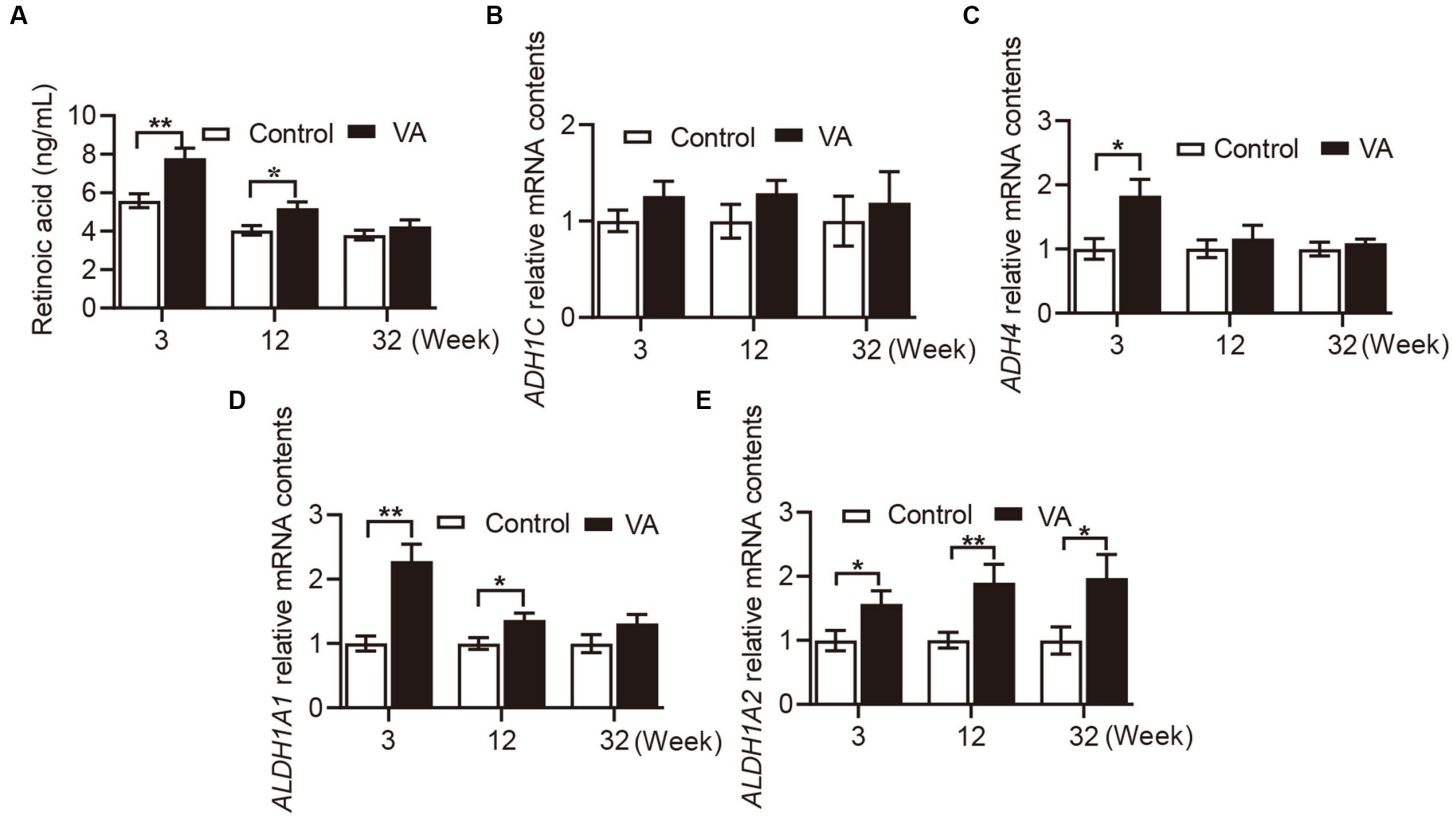
Effects of vitamin A injection on RA content in serum and retinol metabolizing enzymes mRNA expression in LD muscle of lambs. **(A)** RA content in serum. **(B)** Relative mRNA of *ADH1C*. **(C)** Relative mRNA of *ADH4*. **(D)** Relative mRNA of *ALDH1A1*. **(E)** Relative mRNA of *ALDH1A2*. (Mean ± SEM; *n* = 8 in each group, *p < 0.05 and **p < 0.01).

As shown in Figure 1A, VA injection significantly elevated RA content in serum of lambs in the 3rd (Figure 1A, *p* < 0.01) and 12th (Figure 1A, *p* < 0.05) weeks. Consistently, the mRNA level of retinol metabolizing enzymes in VA group showed the same trend in LD muscle of lambs. Specifically, there was no difference in *ADH1C* between VA and control groups (Figure 1B, *p* > 0.05), while *ADH4* expression in VA group was higher than that of control group at the age of 3 weeks (Figure 1C, *p* < 0.05). Moreover, VA increased the level of *ALTH1A1* at the age of 3 (Figure 1D, *p* < 0.01) and 12 weeks (Figure 1D, *p* < 0.05), and VA increased the level of *ALTH1A2* at the age of 3, 12 weeks and harvest (Figure 1E, *p* < 0.05).

### Meat quality and nutritional composition in longissimus dorsi muscle

There were no alterations in pH, meat color (L* and b* values), conductivity, shear force, cook loss, and drip loss in LD muscle between VA and control group of lambs (Table 2, *p* > 0.05). However, lambs in VA group exhibited a higher a*_24h_ value than that in the control group (Table 2, *p* < 0.05). Moreover, VA injection had no effects on moisture and ash content of LD muscle (Table 3, *p* > 0.05), but raised the content of crude protein and crude fat (Table 3, *p* < 0.05).

**Table 2:** Effects of vitamin A injection on meat quality of lambs.

Meat quality		Groups		SEM	*p* value
		Control	VA		
pH	45 min	6.55	6.64	0.14	0.55
	24 h	5.55	5.57	0.05	0.76
L*	45 min	27.10	25.82	0.96	0.21
	24 h	33.18	32.46	0.89	0.44
a*	45 min	9.31	10.24	0.74	0.23
	24 h	9.58	10.90	0.54	0.03
b*	45 min	8.34	8.20	0.32	0.66
	24 h	9.54	9.45	0.29	0.77
Conductivity	45 min	1.86	1.91	0.12	0.69
	24 h	2.53	2.31	0.42	0.61
Shear Force (N)		25.18	23.95	3.07	0.70
Cook Loss (%)		36.57	36.99	0.76	0.58
Drip Loss (%)		18.26	17.51	0.02	0.73

VA, Vitamin A.

**Table 3:** Nutritional composition in longissimus dorsi muscle of lambs.

Items	Groups		SEM	*p* value
	Control	VA		
Moisture, %	72.17	72.20	0.01	0.96
CP, %	20.19	21.62	0.66	0.04
EE, %	2.84	3.56	0.22	0.01
Ash, %	4.65	4.62	0.12	0.83

VA, Vitamin A; CP, crude protein; EE, ether extract.

### Glycogen and collagen abundance in longissimus dorsi muscle and hexokinase, lactate dehydrogenase content in serum

**Figure 2:**
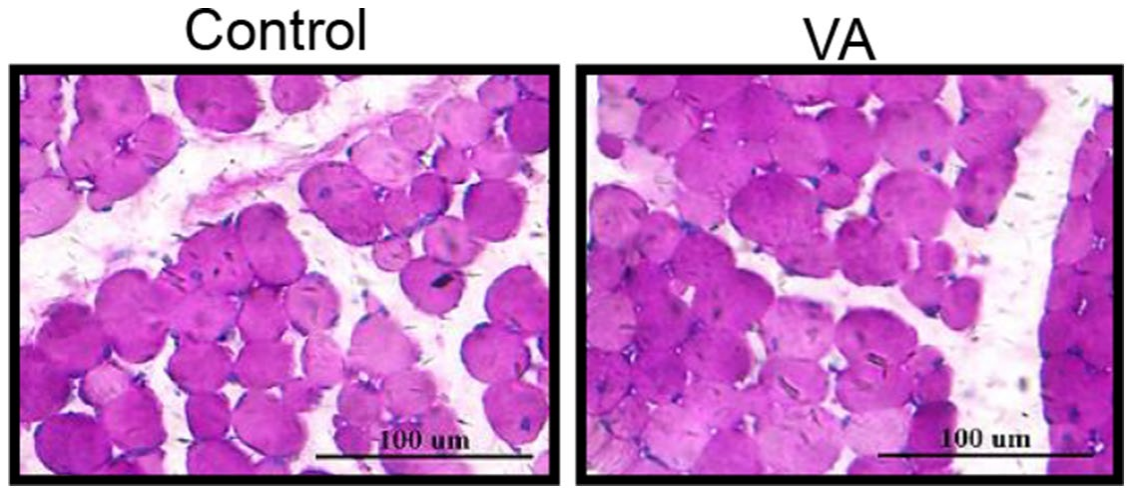
Effects of vitamin A injection on glycogen content in LD muscle of lambs. Glycogen PAS staining of LD muscle. (Mean ± SEM; *n* = 8 in each group).

**Figure 3:**
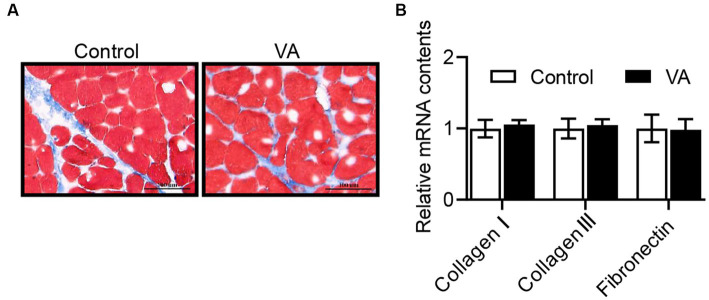
Effects of vitamin A injection on collagen content in LD muscle of lambs. **(A)** Masson staining of LD muscle. **(B)** Relative mRNAs of *collagen I*, *collagen III*, and *fibronectin*. (Mean ± SEM; *n* = 8 in each group).

As shown in Figure 2 and Table 4, VA did not affect the content of glycogen in LD muscle (Figure 2 and Table 4, *p* > 0.05) and the content of HK and LDH in serum (Table 4, *p* > 0.05) of lambs. For collagen content in LD muscle of lambs, there was also no difference between control and VA groups (Figures 3A,B, *p* > 0.05).

**Table 4:** The glycogen content in longissimus dorsi muscle, hexokinase, and lactate dehydrogenase contents in serum of lambs.

Item	Groups		SEM	*p* value
	Control	VA		
Glycogen (mg/g)	4.30	4.65	0.60	0.57
HK (U/L)	38.68	40.76	3.29	0.54
LDH (U/L)	658.25	644.25	19.51	0.49

VA, Vitamin A; HK, hexokinase; LDH, lactate dehydrogenase.

### Amino acid composition in longissimus dorsi muscle

Compared with control group, VA injection had no effects on amino acid composition and total amino acid in LD muscle of lambs (Table 5, *p* > 0.05).

**Table 5:** Amino acid composition in longissimus dorsi muscle of lambs (mg/g).

Amino Acid	Groups		SEM	*p* value
	Control	VA		
Asp	11.02	12.47	1.44	0.34
Glu	20.29	20.74	2.43	0.34
Ser	6.88	7.34	0.65	0.5
Arg	10.64	11.18	0.93	0.58
Gly	6.48	6.73	0.56	0.67
Pro	7.57	7.64	0.51	0.9
Ala	9.97	10.48	0.98	0.62
Thr	5.87	6.26	0.59	0.53
Val	2.57	2.7	0.3	0.67
Met	4.51	4.47	0.38	0.94
Ile	9.67	9.41	0.5	0.61
Leu	11.81	12.46	1.21	0.6
Phe	7.92	8.12	0.62	0.75
His	4.36	4.26	0.33	0.76
Lys	8.97	9.49	1.26	0.69
Tyr	4.67	4.68	0.46	0.99
TAA	133.19	140.43	12.63	0.58

VA, Vitamin A; TAA, total amino acid.

### Antioxidant enzyme activities and malondialdehyde content

**Figure 4:**
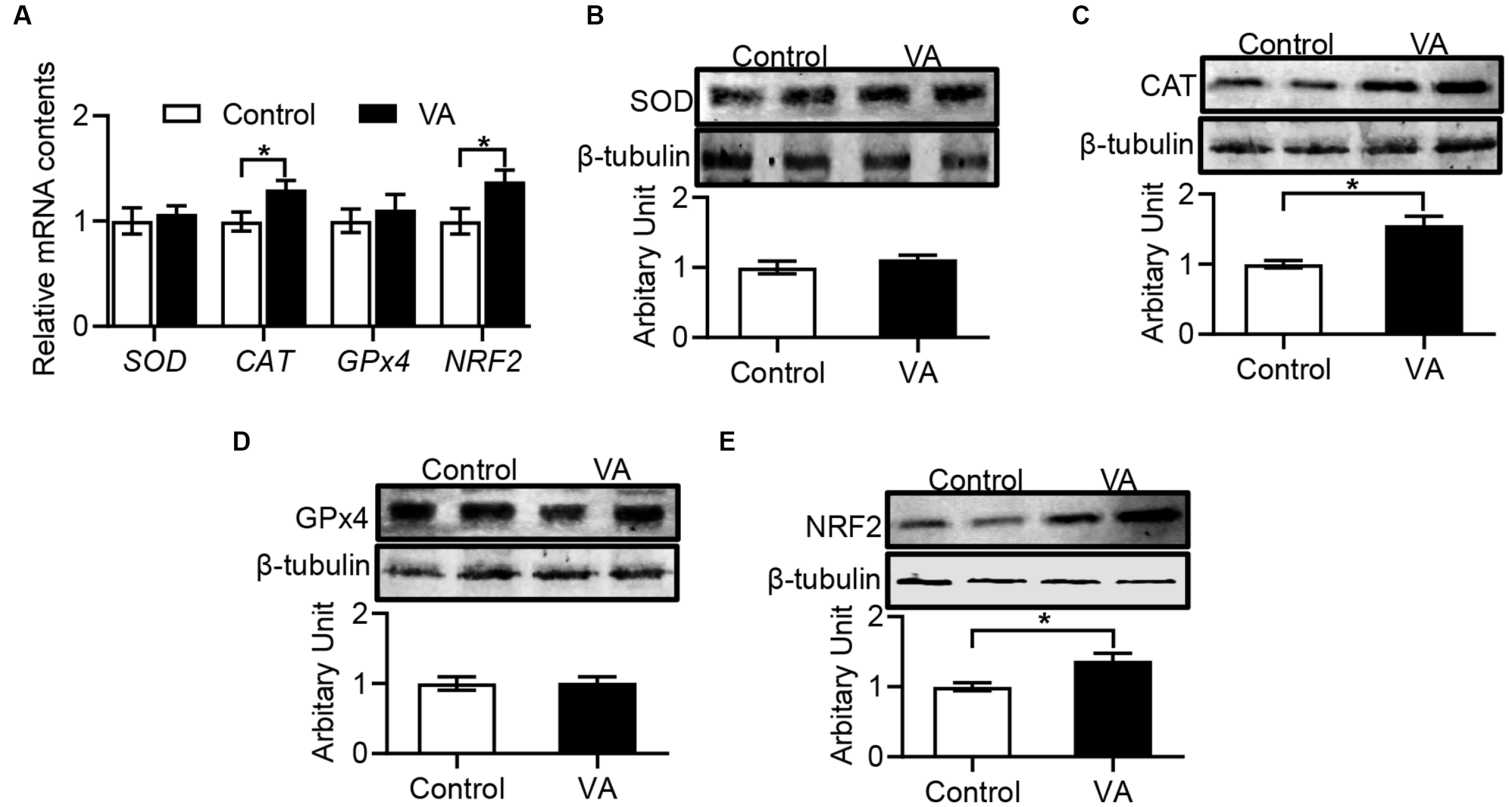
Effects of vitamin A injection on antioxidative activity in LD muscle of lambs. **(A)** Relative mRNAs of *SOD*, *CAT*, *GPx4*, and *NRF2*. **(B)** SOD protein abundance. **(C)** CAT protein abundance. **(D)** GPx4 protein abundance. **(E)** NRF2 protein abundance. (Mean ± SEM; *n* = 8 in each group, *p < 0.05).

Compared with control group, VA increased the CAT activity and reduced the MDA content in LD muscle of lambs (Table 6, *p* < 0.05). Moreover, the data revealed that *CAT* and *NRF2* mRNA expression in LD muscle were higher in VA group, and protein abundance were also significantly elevated (Figures 4A–E, *p* < 0.05).

**Table 6:** Antioxidant enzymes activities and malondialdehyde content in longissimus dorsi muscle of lambs.

Items	Groups		SEM	*p* value
	Control	VA		
T-AOC, U/mg protein	0.19	0.21	0.44	0.65
CAT, U/mg protein	5.93	8.87	1.3	0.04
T-SOD, U/mg protein	37.66	38.04	5.45	0.94
GPx4, U/mg Protein	249.52	295.72	42.66	0.3
MDA, nmol/mg protein	0.5	0.34	0.07	0.04

VA, Vitamin A; T-AOC, total antioxidant capacity; CAT, catalase; T-SOD, total superoxide dismutase; GPx4, glutathione peroxidase 4; MDA, malondialdehyde.

## Discussion

VA has been widely used as a nutritional supplement in the animal husbandry. In the current study, we found that intramuscular injection of VA increased the content of RA in serum of lambs at 3 weeks of age and weaning. RA is known to be the most active metabolite of VA, which mediates the metabolic functions of VA except vision. To explain this phenomenon, we examined the mRNA expression of enzymes that catalyze the synthesis of RA, because RA synthesis is mainly dependent on the catalytic action of alcohol dehydrogenase (ADHS) and aldehyde dehydrogenase (ALDH). However, the data showed that the expression of the enzymes did not seem to be clearly related to RA content, as they exhibited different levels at different stages between control and VA groups, which were consistent with the findings of Harris et al. (19). Indeed, RA has been shown to decrease the sensitivity of chicken embryonic neurons (25, 26), PC12 (27) cells and mesangial cells (28) to oxidative stress. Hence, these results suggested that part of the effect of VA on meat quality might be attributed to the synthesis of high levels of RA.

The pH of the carcass after harvest is dependent on glycogen metabolism in skeletal muscle, and the content of glycogen is positively correlated with the pH value of meat (29). In addition, LDH is a key oxidoreductase in the glycolytic pathway of organisms that catalyzes the formation of lactate acid, and its activity is negatively correlated with pH. In the present study, VA injection did not alter either glycogen in LD muscle or HK and LDH in serum in this trial, indicating that VA did not affect the ultimate pH of lamb meat. Considering that ultimate pH is an important factor affecting meat quality, which is strongly associated with water holding capacity and tenderness (30–32), these data might explain why intramuscular VA injection did not affect holding capacity and tenderness. Indeed, dietary supplementation of natural antioxidants in animals does not affect pH of meat. For example, addition of grape seed extract to the diet had no effect on the final pH of swine muscle (33). In lambs, dietary addition of buckwheat extract also did not affect the pH of the Longissimus thoracis et lumborum muscle (8). Furthermore, intramuscular VA injection did not affect the conductivity, cook loss, and drip loss of LD muscle, which may be attributed to the constant pH value.

Meat color is considered as one of the indicators to judge the health of fresh meat, and flesh redness plays a key role in determining consumers’ decisions (34). In the current study, VA injection significantly increased the LD muscle redness of lambs, as evidenced by an increase in a* value. Similarly, previous studies have shown that supplementation with natural antioxidants increases the redness of meat. For example, feeding pasture abundant in natural antioxidants improved the meat redness (35). Supplementation of finishing pigs with resveratrol or grape seed proanthocyanidin extract improved the pork redness (36, 37). Indeed, meat color is closely related to the level of myoglobin. Kim et al. reported that the increased redness in pork was caused by the high concentration of myoglobin in the muscle (38). When the fresh meat was exposed to air, myoglobin in LD muscle was oxidized to the oxymyoglobin, giving the meat a bright cherry red color, which is what consumers perceive as the color of freshness (39). In addition, meat color is highly correlated with muscle fiber type composition (37). Muscle fibers in adult mammals are divided into four main types: MyHC I, MyHC IIa, MyHC IIb, and MyHC IIx (40, 41). It has been suggested that the higher a* value was always attributed to a high proportion of type I myofiber (38). Consistently, type I myofiber is oxidized muscle fiber with redder flesh color and tend to have higher myoglobin concentrations. However, whether VA injection affected the muscle fiber type composition needs to be further explored.

In beef cattle, intramuscular fat content greatly affected the tenderness and flavor of beef (42, 43), while intramuscular VA injection increased intramuscular fat content through up-regulation of *Zfp423* expression, a key transcription factor regulating adipogenesis (19). Consistently, a higher EE was observed in LD muscle of VA-injected lambs. However, we did not observe the alteration in muscle shear force, implying that VA did not affect lamb meat tenderness. Indeed, meat tenderness is determined by numerous factors, including animal’s breed and age (44), intramuscular collagen content and the length of the sarcomere (45), intramuscular fat content and ultimate pH (46). Interestingly, we observed that VA increased the content of crude protein in LD muscle, in line with a previous study, showing that VA promoted skeletal muscle growth (16).

Lipid oxidation is one of the main causes of meat and meat products spoilage and deterioration (47). The antioxidant system is essential to provide protection against oxidative damage and can be activated by a variety of biologically active substances and antioxidant-related genes (48). Several studies have shown that natural antioxidants can improve meat quality by reducing lipid peroxidation and improving the antioxidant status (49, 50). For example, dietary addition of soy isoflavones improves meat quality by increasing antioxidant capacity in male broilers (51). In pigs, dietary resveratrol supplementation also improves pork quality by increasing antioxidant capacity (37). In this trial, VA treatment significantly increased CAT activity and decreased MDA content in the LD muscle of lambs. Catalase exists in almost all oxygen-exposed organisms (e.g., bacteria, plants and animals), and catalyze the breakdown of hydrogen peroxide into water and oxygen (52). Indeed, catalase have been used to make food wrapping paper to prevent food oxidation in the food industry (53). A previous research has shown that the overexpression of catalase reduced oxidative stress in aging mice (54). The production of MDA has also been used as a bio-marker to assess organismal oxidative stress (55). In addition, Keap1-NRF2 signaling is also important for regulating the antioxidant system, and Keap1 plays a negative regulatory role on activating the antioxidant genes expression such as *CAT*, *SOD1*, and *NRF2* (48, 56). Zhao et al., showed that dietary supplementation of tartary buckwheat extract significantly increased the protein abundance of Gpx4 and Nrf2 and decreased the malondialdehyde content in skeletal muscle, thus improving the antioxidant capacity of lamb muscle (8). Xu et al., found that inclusion of grape seed proanthocyanidin extract in feed of finishing pigs affected mRNA expression of antioxidant related genes, including *SOD1*, *CAT*, *GPX1*, *GST*, *Keap1*, and *NRF2* in LD muscle (36). As expected, our data shown that VA injection enhanced the abundances of NRF2 and CAT, suggesting that VA activating the antioxidant system and protecting the body from oxidative damage. Considering that the shelf life is a major economic constraint for the lamb meat industry (57), the improvement of antioxidant potential in VA-injected lambs may extend the lamb meat’s shelf life.

## Conclusion

In summary, intramuscular VA injection in neonatal stage improved the meat quality of lambs, including postmortem a* value, crude protein and crude fat content, and enhanced the antioxidant capacity in LD muscle.

## Data availability statement

The raw data supporting the conclusions of this article will be made available by the authors, without undue reservation.

## Ethics statement

The animal studies were approved by Institutional Animal Care and Use Committee of Shanxi Agricultural University (sxnd202028). The studies were conducted in accordance with the local legislation and institutional requirements. Written informed consent was obtained from the owners for the participation of their animals in this study.

## Author contributions

PS: Conceptualization, Software, Validation, Visualization, Writing – original draft. GH: Data curation, Formal analysis, Writing – original draft. JF: Investigation, Writing – original draft. WZ: Methodology, Writing – original draft. XL: Methodology, Writing – original draft. JZ: Funding acquisition, Project administration, Resources, Supervision, Writing – review & editing.
